# The Unseen Shift: How Partnership Long-term Care Insurance Influences Caregiving Among Older Adults

**DOI:** 10.1093/geronb/gbae168

**Published:** 2024-10-05

**Authors:** Xianhua Zai

**Affiliations:** Department of Labor Demography, Max Planck Institute for Demographic Research, Rostock, Germany; Max Planck–University of Helsinki Center for Social Inequalities in Population Health, Rostock, Germany/Helsinki, Finland

**Keywords:** Dynamic estimates, Family care, Longitudinal data, TWFE

## Abstract

**Objectives:**

Although the partnership long-term care insurance (PLTC) program was designed to mitigate the low uptake of long-term care insurance (LTCI) and reduce Medicaid’s financial burden, research has predominantly focused on its direct impacts, leaving the effects on informal caregiving unexplored. This study aimed to investigate how the program alters the dynamics of family-provided care, leveraging nationally representative data to unveil the broader consequences of informal caregiving arrangements among older individuals.

**Methods:**

Data for this study were sourced from the U.S. Health and Retirement Study (1992–2018) and linked with the timing of the PLTC program implementation across states. The analysis compared individuals exposed to the program with those who were not, employing 2-way-fixed-effects and dynamic models to assess its impact on LTCI coverage and reliance on informal caregiving.

**Results:**

The program positively affected LTCI coverage, increasing insurance uptake among older individuals in the long run. Conversely, a significant negative effect was observed on the receipt of assistance from any helper, indicating a reduced reliance on informal care. This reduction extended specifically to family helpers and children’s assistance with activities of daily living. The analysis suggests that the program effectively reduced the necessity for informal caregiving across several domains.

**Discussion:**

These findings highlight the program’s potential to reshape caregiving dynamics, suggesting the need for policies that balance promoting private insurance uptake with the implications for family caregiving roles. Policymakers should consider both the economic benefits and the social shifts induced by such programs in the long-term care landscape.

Long-term care (LTC) systems vary across countries, but many face similar challenges, such as low participation in long-term care insurance (LTCI) and rising expenditure on LTC services (Brown & Finkelstein, 2008; Cramer & Jensen, 2006; Curry et al., 2009). To address these issues, several countries, including Germany, the Netherlands, Japan, China, and South Korea, have introduced public LTCI programs (Miyawaki et al., 2020; Pei et al., 2024; Yang et al., 2021). These programs help spread the ﬁnancial risk of LTC, covering formal paid in-home or institutional care, which can serve as substitutes or complements to informal care (Bonsang, 2009; Charles & Sevak, 2005; Liu, 2021; Mellor, 2001; Van Houtven & Norton, 2004; Van Houtven et al., 2015). However, there is limited understanding of how these LTCI programs impact caregivers (Chen et al., 2021; Pei et al., 2024). This study seeks to ﬁll this gap by exploring the spillover effects of one LTCI program in the United States on caregivers.

The partnership long-term care insurance (PLTC) program is a strategic initiative that merges private LTCI with state Medicaid programs. Designed to encourage individuals to purchase private LTCI while protecting their assets for Medicaid eligibility, the PLTC program represents a key intersection between private insurance and public welfare (Costa-Font, 2010; Lin & Prince, 2013). Although most research has focused on the program’s effects on LTCI adoption rates and Medicaid’s ﬁnancial burdens (Sun & Webb, 2023), the potential unintended spillover effects on informal caregiving have been largely overlooked. This study aims to address this knowledge gap by using nationally representative data on older Americans, focusing on the PLTC program’s impact on informal care, particularly within families.

## The PLTC Program

Medicaid is the primary program providing LTC services for eligible older Americans, covering both nursing home care and home- and community-based services (HCBS). Nursing home care includes room, board, nursing services, and therapies, whereas HCBS offers assistance with activities of daily living (ADLs), household management, and medical and personal care at home (Zai, 2024b). Medicaid’s LTC eligibility is based on ﬁnancial and functional criteria. Financial eligibility involves state-speciﬁc income and asset limits, with most states setting income limits at 138% of the federal poverty level for nondisabled adults under 65, and asset limits at $2,000 for singles and $3,000 for couples. Functional eligibility is determined by medical necessity and required level of care, assessed using state-speciﬁc criteria (Zai, 2024b). States are required to recover LTC costs from the estate of deceased Medicaid beneﬁciaries. For those ineligible for Medicaid, the median annual cost of a private nursing home room is approximately $108,405, whereas home health aide services cost around $30,125 per year.

To protect older individuals from the high costs of LTC and the need to deplete assets to qualify for Medicaid, the PLTC program collaborates with state governments and private insurers to encourage the purchase of private LTCI (Bergquist et al., 2018; Costa-Font, 2010; Lin & Prince, 2013). The PLTC program’s key feature is the “dollar-for-dollar” asset protection model, which allows policyholders to protect assets equivalent to the insurance payouts from Medicaid’s spend-down requirements. For example, a $400,000 PLTC policy allows the policyholder to apply for Medicaid with an asset limit of $402,000 instead of the usual $2,000 (Bergquist et al., 2018; Lin & Prince, 2013). The program also mandates inﬂation protection for policyholders under 76, ensuring beneﬁts retain purchasing power over time, and provides estate recovery protection, safeguarding assets from state recovery efforts after the policyholder’s death.

Long-term care insurance policy costs vary based on factors like the insurer, age, marital status, gender, and coverage level (Brown & Finkelstein, 2008). In 2023, the average annual premium for a $165,000 policy for a 55-year-old unmarried male was approximately $900, whereas a single woman of the same age paid around $1,500. Costs increase with age, with a 65-year-old male paying around $1,700 annually, and a female $2,700. For a 65-year-old couple, the combined annual premium is approximately $3,750, demonstrating the variability in LTCI costs (Brown & Finkelstein, 2008).

The PLTC program was initially launched as a pilot in four states: California, Connecticut, Indiana, and New York, during the late 1980s and early 1990s (Bergquist et al., 2018; Costa-Font, 2010). The success and learnings from these pilot programs led to the expansion of PLTC programs across other states. Following the Deﬁcit Reduction Act of 2005, which encouraged broader adoption by offering states the option to establish their own PLTC programs, the rollout expanded signiﬁcantly. This federal endorsement paved the way for a standardized framework, allowing other states to design and implement their PLTC programs while adhering to certain federal requirements (Bergquist et al., 2018). As a result, the availability of PLTC programs varies by state, with each state determining its own launch date, speciﬁc program features, and eligibility criteria. The staggered implementation across states provides a unique landscape for analyzing the program’s impacts on LTCI coverage and other potential outcomes, with each state tailoring the program to meet its residents’ needs while aiming to balance private insurance utilization with public Medicaid expenditures (Lin & Prince, 2013).

## PLTC Programs and LTCI Coverage

The PLTC program is a policy initiative designed to make LTCI more accessible and affordable by offering Medicaid asset protection to policyholders. This approach incentivizes responsible LTC planning and mitigates the ﬁnancial risks of LTC expenses by integrating private insurance with public safety nets (Curry et al., 2009). However, research has shown mixed results. Lin and Prince (2013) found that the program mainly beneﬁted wealthier individuals, with limited impact on overall LTCI purchase rates. Similarly, Bergquist et al. (2018) reported no signiﬁcant increase in LTCI uptake, though there was a rise in insurance applications, suggesting a possible substitution effect between traditional and partnership insurance contracts. Sun and Webb (2023) theorized that PLTC programs would lead to a modest increase in LTCI coverage, primarily among those already inclined to purchase non-partnership policies, with potential costs to Medicaid exceeding the expected savings.

## LTCI Coverage and Family Help

Recent studies have focused on the complex dynamics between LTCI coverage and family-provided care, revealing how LTCI inﬂuences decisions and caregiving patterns within families. This research highlights the nuanced effects of LTCI on family dynamics and the broader implications for informal care. Tsutsui et al. (2014) examined Japan’s LTCI system, introduced in 2000, and its impact on family caregiving and ﬁlial obligations. The study found a decline in the perceived duty to provide care among coresident family caregivers, particularly daughters-in-law, indicating a shift in caregiving roles. Fu et al. (2017) further analyzed the system’s spillover effects on family caregivers’ labor market participation, revealing signiﬁcant positive impacts, with variations by gender and age. Miyawaki et al. (2020) investigated the impact of reduced access to formal LTC in Japan following the 2006 reform of the LTCI system. The study found that decreased access led to increased informal caregiving hours, reduced use of formal LTC services, and a noticeable decline in caregiver health. Stabile et al. (2006) studied caregiving time allocation and home care use in Canada, ﬁnding that increased public ﬁnancing for home care led to higher service utilization, reduced informal caregiving, and improved health outcomes. This suggests that public funding can shift caregiving from informal to formal sectors, positively affecting health. Coe et al. (2023) examined the impact of LTCI on informal care in the United States, ﬁnding no signiﬁcant reduction in informal care over 8 years but observed changes in family dynamics. LTCI coverage reduced expectations of parental care from children, led to less coresidence, and increased labor market engagement among adult children, highlighting economic spillovers of LTCI on families. Korfhage and Fischer-Weckemann (2024) analyzed Germany’s public LTCI system, focusing on the economic impact of informal caregiving. The study showed that without public LTCI, informal caregiving signiﬁcantly reduced labor market participation, leading to lower lifetime earnings and diminished future pension beneﬁts for caregivers. Ning et al. (2024) investigated the effects of China’s LTCI scheme, introduced in 2016, on informal care use across income groups. The study found that LTCI signiﬁcantly reduced reliance on family care among middle-income older individuals but had no signiﬁcant impact on those with low or high incomes. Pei et al. (2024) further explored China’s LTCI, ﬁnding that it signiﬁcantly reduced the care burden and increased labor market participation among informal caregivers, particularly those caring for low-income older individuals or those from farming and informal work sectors.

Although existing studies have shown that LTCI inﬂuences family care, this study’s novelty lies in examining long-term impacts. Understanding these effects is crucial, as they reveal sustained behavioral and economic responses of families and caregivers to LTCI over time, beyond immediate adjustments—an important consideration for policymakers designing effective LTCI policies. The study also explores the dynamic, long-term effects of LTCI on speciﬁc caregiver groups and care types (ADL and instrumental ADL [IADL]), an area often overlooked in the literature. By examining these differentiated impacts, it provides insights into how LTCI affects care provision within families. Additionally, this study addresses the spillover effects of the PLTC program on informal caregivers, a critical aspect often ignored. Although previous research has focused on the short-term impact of PLTC on LTCI coverage, the role of informal caregivers has been largely overlooked. This study highlights how PLTC inﬂuences informal caregiving, offering a more comprehensive evaluation of its effectiveness and potential areas for policy improvement.

## Theoretical Framework for Impact of LTCI on Informal Care

The introduction of LTCI systems represents a signiﬁcant shift in how societies address the needs of aging populations. The implementation of LTCI has far-reaching implications for informal care, which traditionally relies on familial networks and deeply ingrained norms of ﬁlial obligation. This framework explores how LTCI inﬂuences informal care dynamics by considering changes in kinship norms and the interplay between formal and informal care.

### Evolution of Filial Norms and Kinship Obligations

Filial obligation, the duty felt by individuals to care for aging parents or relatives, is a central component of informal care. These norms, however, are not static; they evolve over the life course and are shaped by broader societal changes, including the introduction of LTCI (Tsutsui et al., 2014). The implementation of LTCI increases the role of formal care services, which can lead to a reevaluation of traditional ﬁlial obligations. As societal involvement in adult care grows, individuals may perceive a decreased need for personal involvement, especially in domains where LTCI services are most comprehensive, such as ADLs (Tsutsui et al., 2014).

This shift in perception is particularly pronounced among different family members, depending on their role within the caregiving hierarchy. For instance, primary caregivers, such as family members or children, might experience a more signiﬁcant reduction in their perceived obligations, particularly in physical caregiving, due to the relief provided by LTCI. The extent of this change varies across different dimensions of ﬁlial responsibilities, including physical, emotional, and ﬁnancial support, highlighting the complex interplay between LTCI and kinship obligations (Tsutsui et al., 2014).

### Substitution and Complementarity Between Formal and Informal Care

The relationship between formal care provided by LTCI and informal care by family members can also be understood through the lenses of substitution and complementarity. In some cases, formal care services under LTCI can act as substitutes for informal care. For example, the availability of LTCI services may reduce the reliance on informal care, delaying the need for more intensive services (Coe et al., 2023). This substitution effect is more pronounced in areas where LTCI covers low-skilled care tasks that family members might otherwise have provided (Bonsang, 2009).

However, the interaction between formal and informal care is not always straightforward. As care needs increase, particularly with higher levels of disability, the burden of caregiving can exceed what formal care alone can manage, necessitating a complementary relationship between formal and informal care (Bonsang, 2009). In such cases, LTCI does not replace informal care but rather works alongside it, with both forms of care adjusting to the changing needs of the care recipient. This dual relationship underscores the complexity of care dynamics, where formal and informal care can either substitute for or complement each other depending on the context.

## The Current Study

Leveraging data from the U.S. Health and Retirement Study (HRS), this research examined the effects of PLTC program implementations on the uptake rates of LTCI and the subsequent reliance on informal care, including assistance from family members and relatives. Speciﬁcally, family helpers are identiﬁed as spouses, children, or the spouses of children. Drawing from an extensive review of the scientiﬁc literature and the theoretical frameworks explored previously, the following hypotheses are proposed for investigation:

Hypothesis 1: States that have implemented PLTC programs will see higher LTCI coverage rates among older individuals compared to those in states without such programs.

Hypothesis 2: In states with PLTC programs, older individuals will exhibit a reduced dependency on informal caregivers.

Hypothesis 3: Within the context of PLTC program states, there will be a notable decrease in the need for informal care from family and children helpers among older individuals.

The ﬁrst hypothesis focuses on LTCI uptake because it serves as a crucial preliminary step in examining the subsequent effects on informal care outcomes, as outlined in the theoretical framework. Additionally, this focus allows for a comparison with previous literature, providing insights into both the dynamic and long-term impacts of the PLTC program.

## Method

### Data Source and Study Sample

This research utilized data from the HRS in the period 1992–2018, a nationally representative longitudinal study of Americans aged 51 and older, with biennial surveys since 1992. The HRS includes various birth cohorts, integrated upon reaching eligibility age, with the original HRS cohort tracked from the start (Sonnega et al., 2014). The HRS collects detailed data on demographics, LTCI coverage, health status, family caregiving, and demographic information about family members, including children.

The PLTC program data set provides detailed information on the timing of PLTC program implementation across states, allowing analysis of its effects on individual behaviors and outcomes. By linking this data set with respondents’ state of residence from the HRS, researchers can create a binary policy variable indicating PLTC exposure based on policy adoption relative to HRS survey waves (Costa-Font, 2010; Lin & Prince, 2013).

To examine the PLTC program’s impact on LTCI coverage and informal caregiving, HRS respondents were selected based on their eligibility for the PLTC program at its inception, focusing on individuals under 65 who had no ADL limitations during relevant HRS cycles. Respondents without data on LTCI coverage and caregiving assistance were excluded, resulting in a ﬁnal sample of approximately 60,000 observations from 11,000 respondents.

## Measures

### Dependent Variables

The primary dependent variable was LTCI possession. The HRS asked respondents, “Not including government programs, do you currently hold any long-term care insurance that explicitly provides coverage for nursing home care extending beyond a year or for any aspect of personal or medical care in your residence?” Responses of “Yes” were coded as 1, and “No” responses as 0. Note that the LTCI question in the HRS does not differentiate between traditional LTCI and PLTC policies, limiting the exploration of potential substitution effects between PLTC and non-PLTC insurance (Lin & Prince, 2013).

The secondary dependent variables focused on “helpers,” individuals who provided informal or formal caregiving to HRS participants, often family members supporting daily living and well-being. The HRS gathered data on the presence and extent of such help through comprehensive questionnaires. Participants were asked about assistance with personal care (e.g., bathing and dressing) and instrumental support (e.g., household tasks and transportation; Zai, 2024a). For those reporting help, the HRS inquired about the duration and frequency of assistance in the past month, with questions such as, “In the last month, how many days did you receive help from a caregiver?” and “How many hours daily did your helper assist with personal care tasks?” This data was used to create indicator outcomes reﬂecting whether participants received any help, measured by days or hours.

The HRS also tracked contributions from children, including the number of children helping, and the extent of their assistance in days and hours. Detailed information was collected on the type of help provided, including ADLs (e.g., bathing and dressing) and IADLs (e.g., meal preparation and medication management). Additionally, the survey explored ﬁnancial support from children, such as assistance with living expenses or medical costs.

### Independent Variables and Covariates

The primary independent variable was a binary indicator for the presence of the PLTC program in the respondent’s state at the time of their HRS interview. To account for unobserved factors that might correlate with LTCI coverage and informal care, the analysis used ﬁxed effects for year, state, and individual. Time-varying individual-level covariates, including age, age squared, marital status, number of children, and income, were also included.

### Analytic Strategy

Descriptive statistics were provided for the dependent variable and covariates, comparing individuals exposed to the PLTC program (PLTC = 1) with those not exposed (PLTC = 0). To examine the association between the PLTC program and LTCI coverage (Hypothesis 1), assistance from any helpers (Hypothesis 2), and speciﬁcally family and children help (Hypothesis 3), two-way-ﬁxed-effect models were used. These models included year ﬁxed effects to control for nationwide economic shifts, state-ﬁxed effects to adjust for demographic and policy variations, and individual-ﬁxed effects to account for personal preferences regarding aging and caregiving arrangements.

The analysis utilized two-way fixed effect (TWFE) estimation techniques within Stata 17. The identifying assumption is that the timing and location of PLTC implementation are plausibly random. The variation used for identiﬁcation arises from the implementation of the PLTC policy across different states and over time, with the assumption that these variations are not correlated with unobserved individual-state-year factors (Lin and Prince 2013). Therefore, the preferred speciﬁcation is an individual-state-year ﬁxed effects model. To test the parallel trends assumption, which posits that the outcomes of interest across PLTC implementation groups would have followed similar trends in the absence of the policy, an event-study approach was adopted to illustrate the temporal dynamics of outcome changes, with a speciﬁc pre-period serving as the benchmark window. Standard errors were clustered at the state level, with signiﬁcance thresholds denoted by *p < .05, **p < .01, ***p < .001.

## Results

In states with PLTC programs, LTCI coverage was approximately 10%, compared to 8% in states without such programs (see Table 1). The proportion of HRS participants requiring any help was about 2% in PLTC states, lower than the 3% in non-PLTC states. Assistance with ADLs from family and children was similar across both groups, at about 2% and nearly negligible, respectively. On average, individuals in PLTC states were 60 years old, compared to 53 years in non-PLTC states. Approximately 73% of those in PLTC states were married, compared to 83% in non-PLTC states. Non-PLTC participants typically had around three children, whereas those in PLTC states had about two. Annual income levels were comparable across both groups, averaging around $30,000.

**Table 1.** Descriptive Statistics of Main Variables by PLTC Status in 1992–2018

Variable	PLTC = 0			PLTC = 1		
	*N*	Mean	*SD*	*N*	Mean	*SD*
*Dependent variables*						
LTCI coverage	12,806	0.08	0.27	50,228	0.10	0.31
Any help	11,144	0.03	0.16	50,299	0.02	0.14
Family help	11,144	0.02	0.15	50,299	0.02	0.14
Children help with ADL	10,352	0.00	0.05	45,747	0.00	0.01
*Covariates*						
Age	13,095	53.44	5.47	51,346	59.96	7.88
Marital status (%)	13,087	82.75		51,300	72.86	
Number of children	13,095	3.01	1.93	51,346	2.90	1.95
Income	13,095	29,576	40,745	31,346	30,440	64,236

*Note:* ADL = activities of daily living; LTCI = long-term care insurance; PLTC = partnership long-term care insurance. The table reports descriptive statistics of the working sample of HRS individuals who are age eligible (less than 65) and health eligible (no ADL limitations) when the PLTC program was introduced. The preferred speciﬁcation included year, state, and individual ﬁxed effects where race/ethnicity, gender, place of birth, and religious preference indicators are omitted due to individual ﬁxed effects.

Table 2 shows the impacts of the PLTC program on LTCI coverage (Panel A) and on receiving any help (Panel B). Each column represents a different model speciﬁcation. The ﬁrst column includes year and PLTC-expansion-group ﬁxed effects, grouping states by the year they adopted the PLTC program. The second column adds covariates such as age, age squared, race/ethnicity, gender, marital status, census region, place of birth, religious preference, number of children, and income. The third column replaces PLTC-expansion-group ﬁxed effects with state ﬁxed effects. The fourth column adds individual ﬁxed effects, and the ﬁfth column includes linear time trends.

**Table 2.** Estimates of the PLTC Implementation on LTCI Coverage and Help

Variable	(1)	(2)	(3)	(4)	(5)
Panel A: Long-term care insurance coverage					
Post-PLTC implementation	0.019^**^ (0.006)	0.008 (0.006)	0.007 (0.006)	0.011 (0.006)	0.012 (0.006)
Mean of dependent variable	0.09	0.09	0.09	0.09	0.09
Number of individuals				11,152	11,152
Observations	63,034	62,635	62,634	61,319	61,319
Panel B: Receiving help from any helpers					
Post-PLTC implementation	−0.015^***^ (0.003)	−0.015^***^ (0.003)	−0.016^***^ (0.003)	−0.011^**^ (0.004)	−0.012^***^ (0.003)
Mean of dependent variable	0.13	0.13	0.13	0.13	0.13
Number of individuals				11,154	11,154
Observations	61,443	61,041	61,040	59,750	59,750
Year ﬁxed effects	Y	Y	Y	Y	Y
PLTC-expansion-group ﬁxed effects	Y	Y			
Covariates		Y	Y	Y	Y
State ﬁxed effects			Y	Y	Y
Individual ﬁxed effects				Y	Y
PLTC-expansion-group linear time trends					Y

*Note*: ADL = activities of daily living; LTCI = long-term care insurance; PLTC = partnership long-term care insurance. The table reports the effect of the PLTC implementation on LTCI coverage in Panel A and ever receiving any help in Panel B using the working sample of HRS individuals who are age eligible (less than 65) and health eligible (no ADL limitations) when the PLTC program was introduced. Column (1) shows the estimates without covariates; column (2) adjusts for age, age squared, race/ethnicity, gender, marital status, census region of residence, place of birth, religious preference, number of children, and income for individuals. In column (3) the PLTC-expansion-group ﬁxed effects are replaced by state ﬁxed effects; column (4) adds the individual ﬁxed effects (the preferred speciﬁcation), race/ethnicity, gender, place of birth, and religious preference indicators are omitted due to individual ﬁxed effects; and column (5) includes linear time trends estimated at the PLTC-expansion-group level. Standard errors are clustered at the state level.

^*^ *p < *.05.

^**^ *p* < .01.

^***^ *p* < .001.

**Figure 1.**
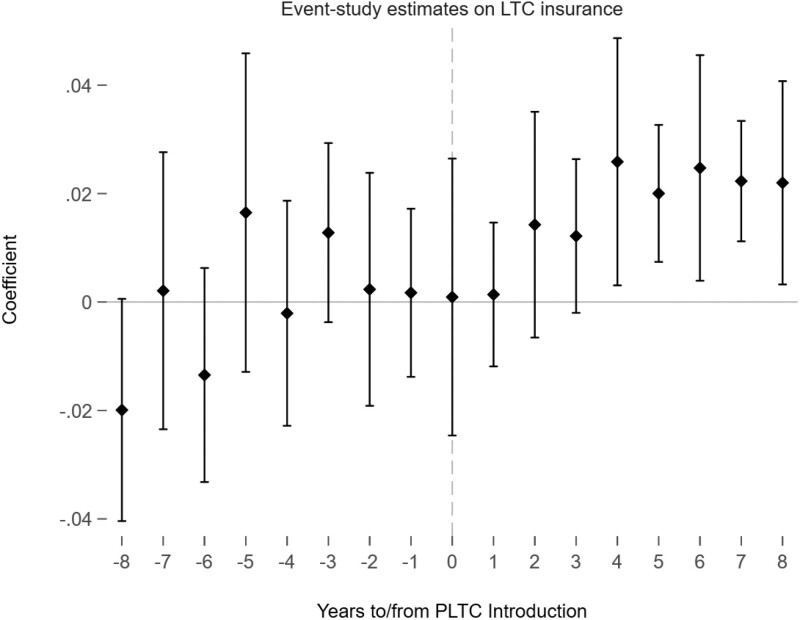
Event-study estimates of the PLTC program on LTC insurance. PLTC = partnership long-term care insurance; LTCI = long-term care. This graph draws the effect with 95 conﬁdence intervals of the PLTC implementation on long-term care insurance coverage using the working sample of Health and Retirement Study (HRS) individuals who are age eligible (less than 65) and health eligible (no ADL limitations) when the PLTC program was introduced. The reference window is all the eight pre-periods before the PLTC introduction. The model adjusts for year-ﬁxed effects and state-ﬁxed effects.

The analysis, as shown in column (4) of Panel A, highlighted the preferred model speciﬁcation. It indicated that the implementation of PLTC increased the probability of LTCI coverage by 1.1 percentage points; however, this effect was not statistically signiﬁcant at the 5% level. To examine the dynamic and long-term effects, Figure 1 illustrated the impact of PLTC on LTCI coverage, showing changes up to 8 years before and after the program’s introduction. The results for the years preceding the program revealed minor, nonsigniﬁcant pre-trend variations, supporting the validity of the parallel trends assumption. Post-implementation, there were positive and statistically signiﬁcant effects beginning 3 years after the program’s introduction, continuing for up to 8 years. The immediate post-implementation estimates were not statistically signiﬁcant, suggesting that it takes time for individuals to respond to the policy. This gradual response also explains the overall nonsigniﬁcance at the 5% level, partially supporting Hypothesis 1 in the long run.

**Figure 2.**
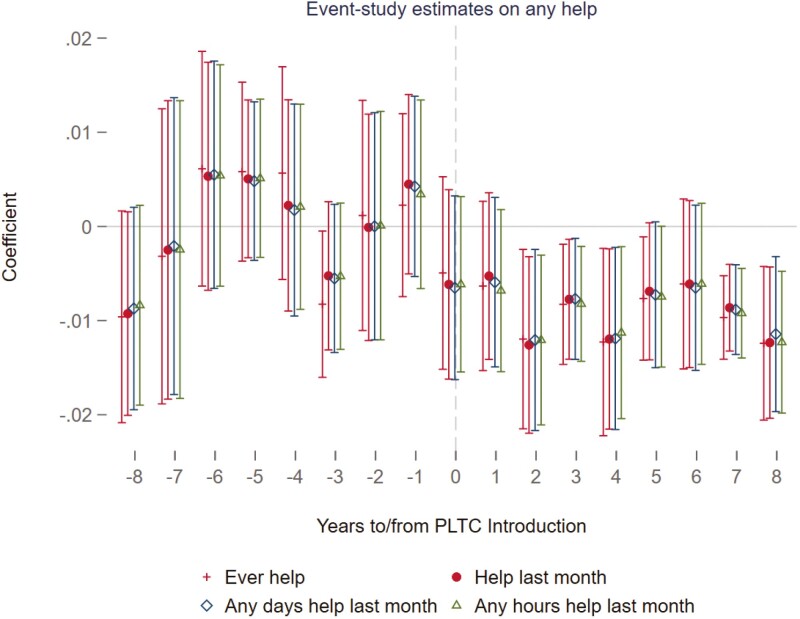
Event-study estimates of the PLTC program on receiving any help. This graph draws the effect with 95 conﬁdence intervals of the PLTC implementation on receiving any help using the working sample of HRS individuals who are age eligible (less than 65) and health eligible (no ADL limitations) when the PLTC program was introduced. The model marked with a red cross uses the dependent variable of the respondent receiving help from any individuals, the red circle uses the variable of receiving any help in the last month, the blue square marks the respondent receiving any help measured in days over the past month, and the green triangle denotes any help measured in hours in the last month. The reference window is all the eight pre-periods before the PLTC introduction. All models adjust for year-ﬁxed effects and state-ﬁxed effects.

**Figure 3.**
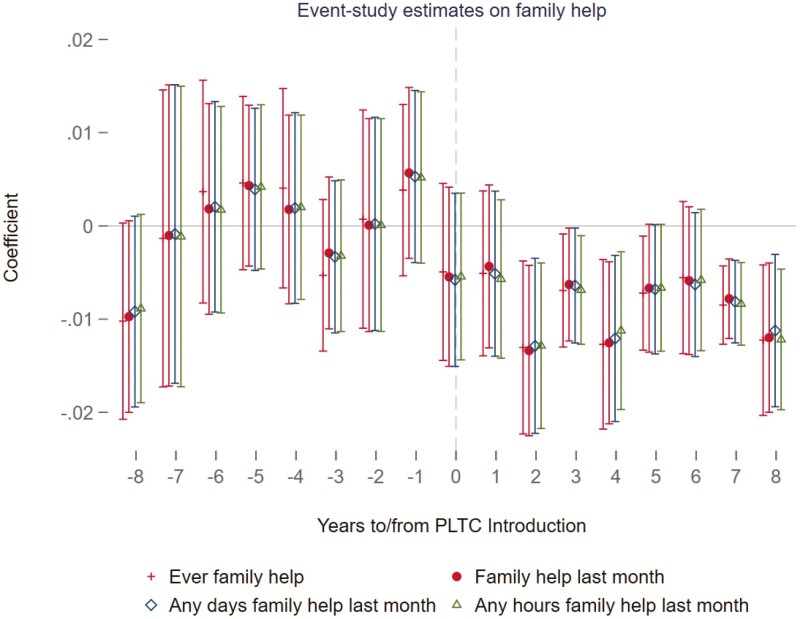
Event-study estimates of the PLTC program on family help. This graph draws the effect with 95 conﬁdence intervals of the PLTC implementation on receiving any help from family members using the working sample of HRS individuals who are age eligible (less than 65) and health eligible (no ADL limitations) when the PLTC program was introduced. The model marked with a red cross uses the dependent variable of the respondent receiving any help from family, the red circle uses the variable of receiving any help from family last month, the blue square marks the respondent receiving any help measured in days, and the green triangle, any help measured in hours from family last month. The reference window is all the eight pre-periods before the PLTC introduction. All models adjust for year-ﬁxed effects and state-ﬁxed effects.

Panel B of Table 2 shows that, following the implementation of PLTC, the probability of receiving help from any caregivers decreased by 1.1 percentage points, which is statistically signiﬁcant at the 1% level according to the individual-state-year ﬁxed effects model in column (4). In contrast, models that included only year and PLTC-expansion-group ﬁxed effects (column (1)) showed larger effects: a 1.9 percentage point increase in LTCI coverage (signiﬁcant at the 1% level) and a 1.5 percentage point reduction in the probability of receiving help (signiﬁcant at the 0.1% level). This suggests that broader ﬁxed effects models, which do not account for detailed individual or trend variations, indicate more pronounced impacts. To delve deeper into the immediate impact of the PLTC program on the provision of help, the study reported estimates from different models for assistance received in the last month by HRS respondents. This was measured across various dimensions: any help received (Supplementary Table S1), any days of help (Supplementary Table S2), and any hours of help (Supplementary Table S3) within the last month. The ﬁndings consistently indicated that the PLTC program signiﬁcantly reduced the probability of receiving help in the last month by 1.1 percentage points, with statistical signiﬁcance at the 1% level. Furthermore, these results were stable across different model speciﬁcations, suggesting that the PLTC program signiﬁcantly lowered individuals’ dependency on receiving assistance. Figure 2 illustrates the dynamic impact of the PLTC program on various forms of combined help, showing a consistent and decreasing effect on the receipt of help over an 8-year period following the program’s implementation. The trend before the program’s introduction was negligible and not statistically signiﬁcant, supporting the parallel trends assumption and highlighting the targeted effect of the PLTC in reducing the need for help among older individuals. The reduction in help began to emerge in the short run, became signiﬁcant in the third year, and remained robust for at least 8 years post-implementation. This evidence supports Hypothesis 2. Supplementary Table S4 elaborated on the impact of the PLTC program on assistance from family helpers, utilizing the preferred model speciﬁcation. The presence of a PLTC program in an individual’s state was found to signiﬁcantly decrease the likelihood of receiving help from any family helpers ever by 1.0 percentage point, with statistical signiﬁcance at the 5% level, diminish the probability of receiving any family help in the previous month by 0.9 percentage point at the 5% level, lower the probability of receiving help, measured in both days and hours, from family members within the last month by similar magnitudes. Conversely, Supplementary Table S5 evaluated the PLTC’s inﬂuence on nonfamily support, revealing much smaller and nonsigniﬁcant ﬁndings across metrics of help received in the last month (β = −0.002, 95% CI = −0.004, 0.001). Figure 3 also depicted a consistent decreasing trend in the receipt of help from family members, further demonstrating the targeted effect of the PLTC program in reducing dependency on family-provided assistance in the short and long run. Hypothesis 3 on family help is supported.

Supplementary Table S6 further detailed the speciﬁc impacts of the PLTC program on assistance provided by children. The availability of a PLTC program was linked to a decrease in help from children, though the result was statistically insigniﬁcant (β = −0.001, *p* = .465). The inﬂuence on children’s assistance with ADL was a 0.2 percentage point decrease, signiﬁcant at the 5% level. Conversely, the effect on IADLs was minimal and not signiﬁcant (β = 0.000, 95% CI = −0.002, 0.003). The impact on children’s assistance with money management was not signiﬁcant (β = −0.001, 95% CI = −0.003, 0.001),and the effect on health care costs was positive but not statistically signiﬁcant (β = 0.001, 95% CI = −0.004, 0.005). Similarly, effects on any transfers from children (β = 0.005, 95% CI = −0.005, 0.015) and any children making transfers (β = 0.005, 95% CI = −0.004, 0.014) were positive but statistically insigniﬁcant. Supplementary Figure S1 illustrated the speciﬁc forms of assistance from children, particularly focusing on ADLs, IADLs, and ﬁnancial transfers, in a dynamic event-study format. The results highlighted a consistent reduction in ADL assistance provided by children in the period following the implementation of the PLTC program. Hypothesis 3 on children help is partially supported.

## Discussion

This study presents ﬁndings on the impact of the PLTC program on LTCI coverage and informal caregiving patterns. By analyzing data from the HRS spanning from 1992 to 2018, the results show a positive effect of the PLTC program on the uptake of LTCI. The magnitude of the positive effect on LTCI coverage is larger than previously reported, becoming statistically signiﬁcant four periods after PLTC implementation and remaining consistent 8 years post-implementation. However, the overall nonsigniﬁcance of these estimates aligns with existing ﬁndings. Several factors might explain the differences from previous studies. First, it takes time for individuals to respond to the PLTC program and decide to purchase LTCI. A shorter observation period, as used in Lin and Prince (2013), might not capture signiﬁcant effects. Indeed, the dynamic analysis shows that it takes at least 4 years to see an increase in LTCI uptake. Second, individuals with preexisting conditions are often underwritten from LTCI. This study excludes individuals with ADL limitations from the analysis sample, whereas Lin and Prince (2013) did not. Including unhealthy individuals in their sample could mitigate the overall magnitude of the results, which in this study is larger. Third, this study beneﬁts from the use of individual-ﬁxed effects, leveraging the longitudinal HRS data to adjust for individual-level unobservable characteristics. In contrast, the smaller sample size and lack of adjustments for individual-level observable and unobservable factors in Bergquist et al. (2018) could bias estimates toward zero.

Regarding the impact on informal caregiving, this research serves as a pioneering exploration of the impact of the PLTC program on informal caregiving among older individuals. The ﬁndings reveal that the introduction of the PLTC program reduced the likelihood of informal caregivers providing care, which is consistent with the outcomes observed in other LTC programs (Fu et al., 2017; Pei et al., 2024; Stabile et al., 2006; Tsutsui et al., 2014). The dynamic event-study estimates suggest that this reduction in informal caregiving began to materialize 2 years after the policy was implemented, likely due to the time it takes for older individuals to respond to the policy incentives as they anticipate needing more LTC services. Furthermore, the analysis showed that the reduction in caregiving primarily beneﬁted family members, who typically bear a signiﬁcant burden in providing LTC to older individuals who become dependent (Pei et al., 2024). The decrease in reliance on family members not only alleviates these burdens but also potentially enhances the quality of life for both caregivers and care recipients (Stabile et al., 2006). By reducing caregiving responsibilities, families can maintain their roles without the added strain of caregiving duties, thereby fostering better relationships and overall well-being (Broyles et al., 2016; Pezzin et al., 2008). Moreover, the sustained reduction in informal caregiving, which persisted even 8 years after the PLTC implementation, provides strong evidence of the program’s signiﬁcant spillover effect and its lasting impact on reducing informal care. This ﬁnding is crucial because if the program had only temporarily decreased the informal care burden, with levels eventually returning to their previous state, it would suggest limited effectiveness, particularly given the substantial psychological, physical, and ﬁnancial costs associated with informal caregiving (Amirkhanyan & Wolf 2006; Bom et al., 2019a, 2019b; Kim et al., 2013; Kolodziej et al., 2022; Lee & Tang, 2015; Moussa, 2019; Pavalko & Artis, 1997; Pinquart & Sorensen, 2007; Scharlach, 1994; Schulz et al., 1995; Van Houtven et al., 2019). The long-term reduction in informal caregiving underscores the program’s success in alleviating the caregiving burden over an extended period.

The ﬁndings of this research have signiﬁcant policy implications for LTC programs. The study shows that the PLTC program effectively increased LTCI coverage in the long run. However, underwriting challenges that prevent individuals with preexisting conditions from obtaining LTCI can limit the program’s effectiveness in encouraging LTCI purchase. To address this, policymakers should consider revising the underwriting process to make LTCI more inclusive. Potential solutions include offering alternative coverage options for high-risk individuals, such as state-supported insurance plans, or implementing more ﬂexible underwriting criteria. By making LTCI more accessible, the PLTC program can more effectively reduce the burden of informal caregiving and extend its beneﬁts to a broader population.

Second, when designing LTC programs, policymakers should consider the household as a key unit in decision-making processes, such as LTCI purchases and the arrangement of LTC services. The ﬁndings on the spillover effect, which show a reduction in informal caregiving by family members, highlight that addressing LTC needs is not merely an individual concern but also a family issue that must be accounted for. Effective LTC policies should recognize that families often play a central role in caregiving decisions and responsibilities. Therefore, policymakers should internalize and leverage family dynamics by providing incentives that encourage families to make collective decisions that align with the program’s goals. This could include offering family-based discounts on LTCI premiums, providing tax incentives for families who purchase LTCI or coordinate care services together, and creating support systems that ease the caregiving burden on families. By aligning LTC programs with the realities of family involvement, policymakers can create more effective and comprehensive solutions that better meet the needs of both the aging population and their caregivers.

### Limitations

Utilizing the HRS to assess the impact of PLTC programs on LTCI coverage and family assistance has several limitations. First, the HRS lacks data on LTCI applications, limiting the analysis of initial interest and potential application barriers (Bergquist et al., 2018). This gap restricts understanding of the factors inﬂuencing LTCI application decisions. Second, the HRS does not provide details on LTCI contract speciﬁcs such as coverage limits, premiums, and beneﬁts, which hinders evaluation of how these features might affect the appeal of PLTC programs and LTCI acquisition decisions. Third, the study cannot examine potential substitution between traditional LTCI and PLTC policies. Because the HRS LTCI question does not differentiate between LTCI types, it is difficult to assess whether individuals are simply switching from traditional LTCI to PLTC policies rather than increasing overall LTCI uptake. Additionally, the HRS’s focus on older Americans limits insights into how PLTC programs affect younger individuals’ decisions to acquire LTCI for future needs. The self-reported nature of HRS data also introduces potential biases due to inaccurate recall or selective reporting, particularly regarding sensitive ﬁnancial information and caregiving dynamics. Finally, the biennial survey structure of the HRS may not capture timely responses to policy changes, as the effects of PLTC programs on LTCI coverage and family assistance may evolve more rapidly than the survey intervals can detect.

## Conclusion

This study enriches the existing body of research by providing some evidence into the unintended impacts of the PLTC program on informal caregiving. The analysis revealed that the PLTC program signiﬁcantly elevated LTCI coverage rates among older individuals who met the age and health criteria during its implementation in the long run. Beyond just enhancing LTCI coverage, participants in the HRS with access to the PLTC program signiﬁcantly diminished their dependence on support, particularly from family members, and eased the caregiving load on children assisting with ADL. These unforeseen inﬂuences on informal care offer new perspectives for assessing the PLTC program’s eﬃcacy and suggest potential policy enhancements focused on asset protection to motivate LTCI purchases.

In summary, the PLTC program has shown potential promise in transforming the landscape of LTCI and modifying patterns of caregiving. Its role in amplifying LTCI coverage and lightening the load of informal caregiving heralds a move towards a more resilient and sustainable system of LTC ﬁnancing. However, the wider economic consequences and the future viability of Medicaid remain subjects for deeper exploration. With the growing need for LTC, forward-thinking policies akin to the PLTC program will be essential in navigating these challenges and fortifying the support network for the aging population and their caregivers.

## Supplementary Material

gbae168_suppl_Supplementary_Tables_S1-S5_Figure_S1

## Data Availability

The public data for the U.S. Health and Retirement Study are available from the official website at https://hrs.isr.umich.edu/, and the restricted HRS data are available once the application is approved from HRS. The policy data are public available.
