# Supplementary material for: The Unseen Shift: How Partnership Long-term Care Insurance Influences Caregiving Among Older Adults
Source: J Gerontol B Psychol Sci Soc Sci. 2024 Oct 5;79(12):gbae168. doi: 10.1093/geronb/gbae168 (PMC11638482; doi:10.1093/geronb/gbae168)
Supplement: gbae168_suppl_Supplementary_Tables_S1-S5_Figure_S1 [file gbae168_suppl_supplementary_tables_s1-s5_figure_s1.docx]

**The Journals of Gerontology, Series B: Psychological Sciences and Social Sciences Supplementary Material: Zai. The Unseen Shift: How Partnership Long-Term Care Insurance Influences Caregiving Among Older Adults**


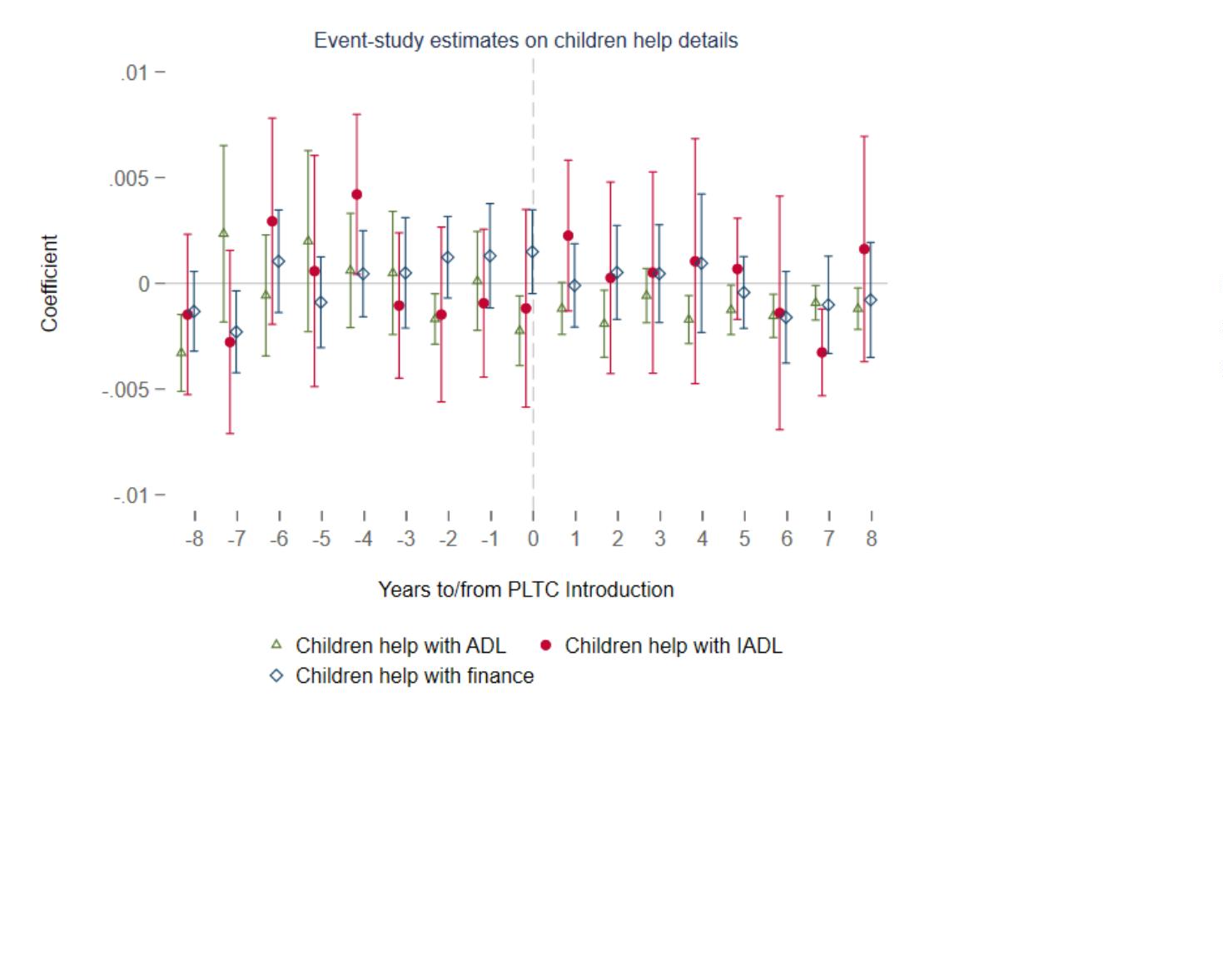


Supplementary Figure 1: Event-study estimates of the PLTC program on children help details

Note. This graph draws the eﬀect with 95 conﬁdence intervals of the PLTC implementation

on receiving any help from children using the working sample of HRS individuals who are age

eligible (less than 65) and health eligible (no ADL limitations) when the PLTC program was

introduced. The model marked with a green triangle uses the dependent variable of children

helping with basic activities of daily living (ADLs), such as bathing, dressing, eating, getting

in and out of bed, and walking across a room; the model labeled with the red circle includes

help with instrumental activities of daily living (IADL), such as using the phone, managing

money, taking medications, shopping for groceries, and preparing hot meals; and the blue

square indicate any children help with money management. The reference window is all the

eight pre-periods before the PLTC introduction. All models adjust for year-ﬁxed eﬀects and

state ﬁxed eﬀects.

Supplementary Table 1: Estimates of the PLTC implementation on receiving any help within the last month

|  | Receiving help from any helpers in the last month | | | | |
| --- | --- | --- | --- | --- | --- |
|  | (1) | (2) | (3) | (4) | (5) |
| Post PLTC implementation | -0.015*** (0.003) | -0.014*** (0.003) | -0.015*** (0.003) | -0.011** (0.004) | -0.012** (0.003) |
| Mean of dependent variable | 0.12 | 0.12 | 0.12 | 0.12 | 0.12 |
| Number of individuals |  |  |  | 11,150 | 11,150 |
| Observations | 61,419 | 61,017 | 61,016 | 59,722 | 59,722 |
| Year ﬁxed eﬀects | Y | Y | Y | Y | Y |
| PLTC-expansion-group ﬁxed eﬀects | Y | Y |  |  |  |
| Covariates |  | Y | Y | Y | Y |
| State ﬁxed eﬀects |  |  | Y | Y | Y |
| Individual ﬁxed eﬀects |  |  |  | Y | Y |
| PLTC-expansion-group linear time trends |  |  |  |  | Y |

Note. The table reports the eﬀect of the PLTC implementation on receiving any help within the last month using the working sample of HRS individuals who are age eligible (less than 65) and health eligible (no ADL limitations) when the PLTC program was introduced. This table reports on the eﬀect of the PLTC implementation on receiving any help within the last month. Column (1) shows the estimates without covariates; column (2) adjusts for age, age squared, race/ethnicity, gender, marital status, census region of residence, place of birth, religious preference, number of children, and income for individuals. In column (3) the PLTC-expansion-group ﬁxed eﬀects are replaced by state ﬁxed eﬀects; column (4) adds the individual ﬁxed eﬀects (the preferred speciﬁcation), race/ethnicity, gender, place of birth, and religious preference indicators are omitted due to individual ﬁxed eﬀects; column (5) includes linear time trends estimated at the PLTC-expansion-group level. Standard errors are clustered at the state level. * p<.05, ** p<.01, *** p<.001.

Supplementary Table 2: Estimates of the PLTC implementation on receiving any days of help

|  | Any days got help last month | | | | |
| --- | --- | --- | --- | --- | --- |
|  | (1) | (2) | (3) | (4) | (5) |
| Post PLTC implementation | -0.015*** (0.003) | -0.014*** (0.003) | -0.015*** (0.003) | -0.011** (0.004) | -0.012*** (0.003) |
| Mean of dependent variable | 0.12 | 0.12 | 0.12 | 0.12 | 0.12 |
| Number of individuals |  |  |  | 11,150 | 11,150 |
| Observations | 61,401 | 60,999 | 60,998 | 59,706 | 59,706 |
| Year ﬁxed eﬀects | Y | Y | Y | Y | Y |
| PLTC-expansion-group ﬁxed eﬀects | Y | Y |  |  |  |
| Covariates |  | Y | Y | Y | Y |
| State ﬁxed eﬀects |  |  | Y | Y | Y |
| Individual ﬁxed eﬀects |  |  |  | Y | Y |
| PLTC-expansion-group linear time trends |  |  |  |  | Y |

Note. The table reports the effect of the PLTC implementation on receiving any help, measured in any days in the last month using the working sample of HRS individuals who are age eligible (less than 65) and health eligible (no ADL limitations) when the PLTC program was introduced. Column (1) shows the estimates without covariates; column (2) adjusts for age, age squared, race/ethnicity, gender, marital status, census region of residence, place of birth, religious preference, number of children, and income for individuals. In column (3) the PLTC-expansion-group fixed effects are replaced by state fixed effects; column (4) adds the individual fixed effects (the preferred specification), race/ethnicity, gender, place of birth, and religious preference indicators are omitted due to individual fixed effects; column (5) includes linear time trends estimated at the PLTC-expansion-group level. Standard errors are clustered at the state level. * p<.05, ** p<.01, *** p<.001.

Supplementary Table 3: Estimates of the PLTC implementation on receiving any hours of help

|  | Any days got help last month | | | | |
| --- | --- | --- | --- | --- | --- |
|  | (1) | (2) | (3) | (4) | (5) |
| Post PLTC implementation | -0.014*** (0.003) | -0.014*** (0.003) | -0.014*** (0.003) | -0.010** (0.004) | -0.013*** (0.003) |
| Mean of dependent variable | 0.11 | 0.11 | 0.11 | 0.11 | 0.11 |
| Number of individuals |  |  |  | 11,146 | 11,146 |
| Observations | 61,368 | 60,966 | 60,965 | 59,670 | 59,670 |
| Year ﬁxed eﬀects | Y | Y | Y | Y | Y |
| PLTC-expansion-group ﬁxed eﬀects | Y | Y |  |  |  |
| Covariates |  | Y | Y | Y | Y |
| State ﬁxed eﬀects |  |  | Y | Y | Y |
| Individual ﬁxed eﬀects |  |  |  | Y | Y |
| PLTC-expansion-group linear time trends |  |  |  |  | Y |

Note. The table reports the effect of the PLTC implementation on receiving any help, measured in any hours in the last month using the working sample of HRS individuals who are age eligible (less than 65) and health eligible (no ADL limitations) when the PLTC program was introduced. Column (1) shows the estimates without covariates; column (2) adjusts for age, age squared, race/ethnicity, gender, marital status, census region of residence, place of birth, religious preference, number of children, and income for individuals. In column (3) the PLTC-expansion-group fixed effects are replaced by state fixed effects; column (4) adds the individual fixed effects (the preferred specification), race/ethnicity, gender, place of birth, and religious preference indicators are omitted due to individual fixed effects; column (5) includes linear time trends estimated at the PLTC-expansion-group level. Standard errors are clustered at the state level. * p<.05, ** p<.01, *** p<.001.

Supplementary Table 4: Estimates of the PLTC implementation on receiving help from family helpers

|  | Help in the last month | | | |
| --- | --- | --- | --- | --- |
|  | (1) | (2) | (3) | (4) |
|  | Ever help | Any help | Any days | Any hours |
| Post PLTC implementation | -0.010* (0.004) | -0.009* (0.004) | -0.009* (0.004) | -0.009* (0.004) |
| Mean of dependent variable | 0.12 | 0.11 | 0.11 | 0.10 |
| Number of individuals | 11,154 | 11,150 | 11,150 | 11,146 |
| Observations | 59,750 | 59,730 | 59,717 | 59,684 |
| R-squared | 0.369 | 0.366 | 0.367 | 0.363 |

Note. The table reports the effect of the PLTC implementation on receiving help from family members using the working sample of HRS individuals who are age eligible (less than 65) and health eligible (no ADL limitations) when the PLTC program was introduced. Column (1) reports the effect of the PLTC implementation on ever receiving any help from family members; column (2) on receiving such help within the last month; column (3) on any help measured in days; column (4) on any help measured in hours that respondents received in the last month from non-family members. All models adjust for year-fixed effects, state fixed effects, individual fixed effects as well as individual time-varying demographics such as age, age squared, marital status, number of children, and income. Race/ethnicity, gender, place of birth, and religious preference indicators are omitted due to individual fixed effects. Standard errors are clustered at the state level. * p<.05, ** p<.01, *** p<.001.

Supplementary Table 5: Estimates of the PLTC implementation on receiving help from non-family members

|  | Help in the last month | | | |
| --- | --- | --- | --- | --- |
|  | (1) | (2) | (3) | (4) |
|  | Ever help | Any help | Any days | Any hours |
| Post PLTC implementation | -0.002 (0.001) | -0.002 (0.001) | -0.002 (0.001) | -0.002 (0.001) |
| Mean of dependent variable | 0.03 | 0.02 | 0.02 | 0.02 |
| Number of individuals | 11,154 | 11,154 | 11,154 | 11,154 |
| Observations | 59,750 | 59,740 | 59,737 | 59,733 |
| R-squared | 0.340 | 0.344 | 0.339 | 0.345 |

Note. The table reports the effect of the PLTC implementation on receiving help from non-family members using the working sample of HRS individuals who are age eligible (less than 65) and health eligible (no ADL limitations) when the PLTC program was introduced. Column (1) reports the effect of the PLTC implementation on receiving any help from non-family helpers; column (2) on receiving such help within the last month; column (3) on any help measured in days; column (4) on any help measured in hours; that respondents receive in the last month from non-family members. The average value of each dependent variable (DV) is shown in the mean row. All models adjust for year-fixed effects, state fixed effects, individual fixed effects as well as individual time-varying demographics such as age, age squared, marital status, number of children, and income. Race/ethnicity, gender, place of birth, and religious preference indicators are omitted due to individual fixed effects. Standard errors are clustered at the state level. * p<.05, ** p<.01, *** p<.001.
